# Immunological profile in a family with nephrogenic diabetes insipidus with a novel 11 kb deletion in AVPR2 and ARHGAP4 genes

**DOI:** 10.1186/1471-2350-9-42

**Published:** 2008-05-20

**Authors:** Masaya Fujimoto, Kohsuke Imai, Kenji Hirata, Reiichi Kashiwagi, Yoichi Morinishi, Katsuhiko Kitazawa, Sei Sasaki, Tadao Arinami, Shigeaki Nonoyama, Emiko Noguchi

**Affiliations:** 1 Department of Medical Genetics, Graduate School of Comprehensive Human Sciences, University of Tsukuba, Ibaraki, Japan; 2 Department of Pediatrics, National defense medical college, Saitama, Japan; 3 Department of pediatrics, Ibaraki children hospital, Ibaraki, Japan; 4 Department of pediatrics, Asahi chyuo hospital, Chiba, Japan; 5 Department of Nephrology, Tokyo Medical and Dental University Graduate School, Tokyo, Japan

## Abstract

**Background:**

Congenital nephrogenic diabetes insipidus (NDI) is characterised by an inability to concentrate urine despite normal or elevated plasma levels of the antidiuretic hormone arginine vasopressin. We report a Japanese extended family with NDI caused by an 11.2-kb deletion that includes the entire *AVPR2 *locus and approximately half of the *Rho GTPase-activating protein 4 *(*ARHGAP4*) locus. ARHGAP4 belongs to the RhoGAP family, Rho GTPases are critical regulators of many cellular activities, such as motility and proliferation which enhances intrinsic GTPase activity.

ARHGAP4 is expressed at high levels in hematopoietic cells, and it has been reported that an NDI patient lacking *AVPR2 *and all of *ARHGAP4 *showed immunodeficiency characterised by a marked reduction in the number of circulating CD3+ cells and almost complete absence of CD8+ cells.

**Methods:**

PCR and sequencing were performed to identify the deleted region in the Japanese NDI patients. Immunological profiles of the NDI patients were analysed by flow cytometry. We also investigated the gene expression profiles of peripheral blood mononuclear cells (PBMC) from NDI patients and healthy controls in microarray technique.

**Results:**

We evaluated subjects (one child and two adults) with 11.2-kb deletion that includes the entire *AVPR2 *locus and approximately half of the *ARHGAP4*. Hematologic tests showed a reduction of CD4+ cells in one adult patient, a reduction in CD8+ cells in the paediatric patient, and a slight reduction in the serum IgG levels in the adult patients, but none of them showed susceptibility to infection. Gene expression profiling of PBMC lacking *ARHGAP4 *revealed that expression of RhoGAP family genes was not influenced greatly by the lack of *ARHGAP4*.

**Conclusion:**

These results suggest that loss of *ARHGAP4 *expression is not compensated for by other family members. ARHGAP4 may play some role in lymphocyte differentiation but partial loss of *ARHGAP4 *does not result in clinical immunodeficiency.

## Background

Maintenance of body fluid volume and composition is essential for proper physiologic function in humans. Under normal conditions, the glomerular filtration rate of the two kidneys is 180 L day^-1^, and up to 90% of the filtrate is reabsorbed in the proximal tubule and descending limb of Henle's loop. The key hormone that regulates reabsorption is the antidiuretic hormone arginine vasopressin (AVP), which is secreted by the posterior pituitary in response to hypovolemia or hypernatremia [1]. AVP is transported by the blood to the kidney and binds to arginine vasopressin receptor 2 (AVPR2), leading to an increase in intracellular cAMP levels via the stimulatory Gs protein and adenylate cyclase, and to subsequent activation of protein kinase A and phosphorylation of aquaporin 2 (AQP2) water channels [2]. This process is necessary for proper reabsorption of the water in the principal cells of the collecting duct under the control of AVPR2 [3].

Congenital nephrogenic diabetes insipidus (NDI) is characterised by an inability to concentrate urine despite a normal or elevated plasma level of AVP. Two genes have been reported to be associated with NDI; X-linked *AVPR2 *[4] and autosomal *AQP2 *[5,6]. The X-linked form of NDI is present in up to 90% of patients. Males with the disease-causing mutation are usually affected, and females heterozygous for the disease-causing mutation show various degrees of penetrance. Skewed X inactivation, which is preferential methylation of the normal allele of the *AVPR2 *gene, can cause NDI in female heterozygotes [7].

To date, 178 *AVPR2 *mutations, including 12 gross deletions [8-13], have been deposited in the BIOBASE database [14]. Large deletions that lead to complete loss of *AVPR2 *and parts of the neighboring genes *ARHGAP4 *[9,11,15] and *L1 cell adhesion molecule *(*L1CAM*) [16] have been reported. *ARHGAP4*, which is a member of the GTPase-activating protein family, is located telomeric to *AVPR2 *and is expressed at a high level in hematopoietic cells. Recently, an NDI patient lacking *AVPR2 *and all of *ARHGAP4 *showed immunodeficiency characterised by a marked reduction in the number of circulating CD3+ cells and almost complete absence of CD8+ cells [17]. Herein, we describe a Japanese extended family with multiple NDI patients lacking the entire *AVPR2 *locus and approximately half of *ARHGAP4*. Although none of the family members with NDI showed clinical signs of immunodeficiency, immunologic profiling showed slight abnormalities.

## Results

### Mutation screening

**Figure 1:**
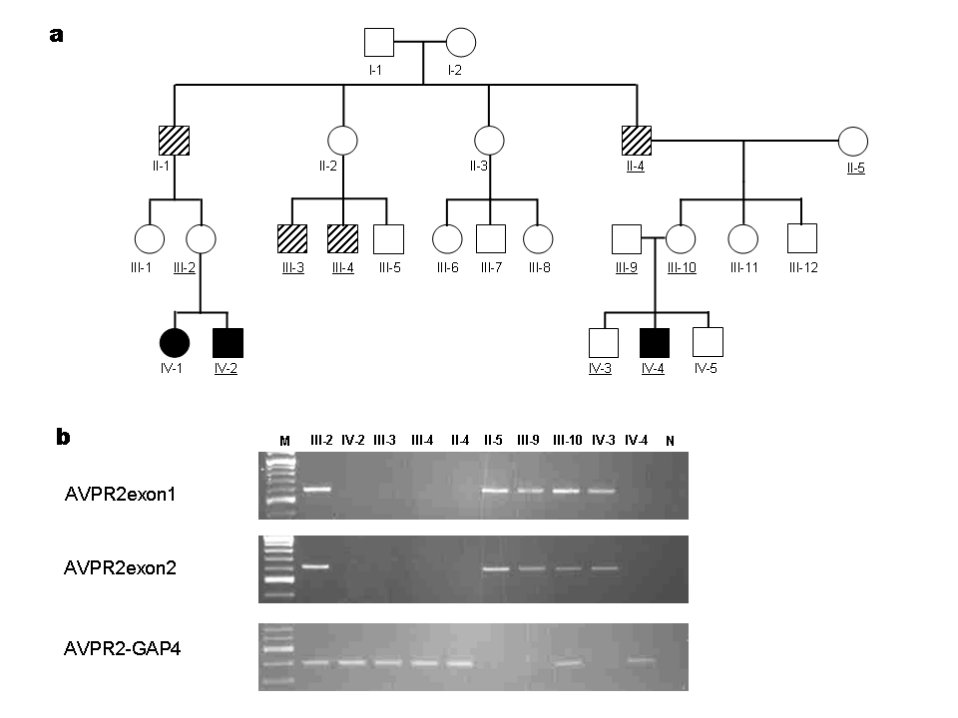
**a; The pedigree of the family with NDI**. A closed pox indicates affected members. Several family members (II-1, II-4, III-3, and III-4, indicated by a diagonal box) have histories of polyuria and polydipsia but have not been diagnosed with NDI. Family members marked with underlines are ones for PCR analysis. b; PCR amplification of the NDI family members. M, 100 bp marker, N, negative control. The regions amplified with the primer pairs (AVPR2exon1, AVPR2exon2, and AVPR2-GAP) are shown in Figure 2.

Two patients (IV-2 and IV-4 in Figure 1) were admitted to the hospital at the age of 2 months with fever of unknown origin. NDI was diagnosed on the basis of clinical symptoms and laboratory findings (dehydration, hypernatremia, and hypotonic urine) and failure to increase urine osmolarity in response to 1-2esamino-8-D-arginine vasopressin (dDAVP) (Table 1). The sister (IV-1) had no history of dehydration, but polyuria and polydipsia were noticed by her family members, and NDI was diagnosed on the basis of laboratory findings at the age of 2 years. None of the patients had any evidence of mental retardation or significant disease other than NDI. The pedigree of the Japanese NDI family is shown in Figure 1. Subjects indicated by a diagonal box (II-1, III-3, III-4, and II-4) have a history of polyuria and polydipsia since childhood, but NDI has not been diagnosed.

**Figure 2:**
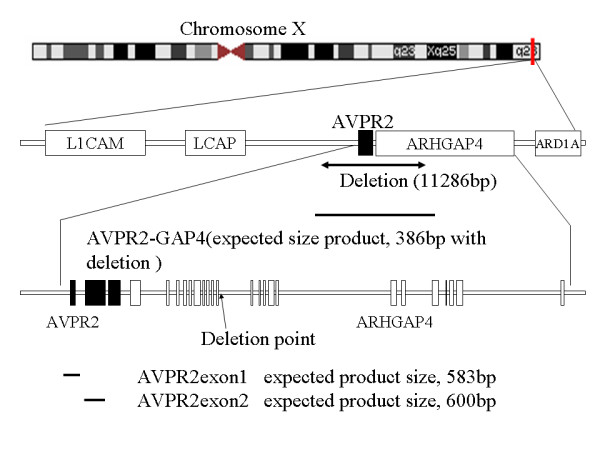
**The physical map and the deleted region in the Japanese NDI family**. 11,286 bp deletion was observed around *AVPR2 *gene. Bold lines indicated amplified regions with primers (AVPR2-GAP, 386 bp with deletion, AVPR2exon1, expected product size 583 bp with no deletion, and AVPR2exon2, expected product size 600 bp with no deletion). 5' deleted point occurred between *LCAP *and *AVPR2*, and 3' deleted point was in the intron 10 of *ARHGAP4*.

**Figure 3:**
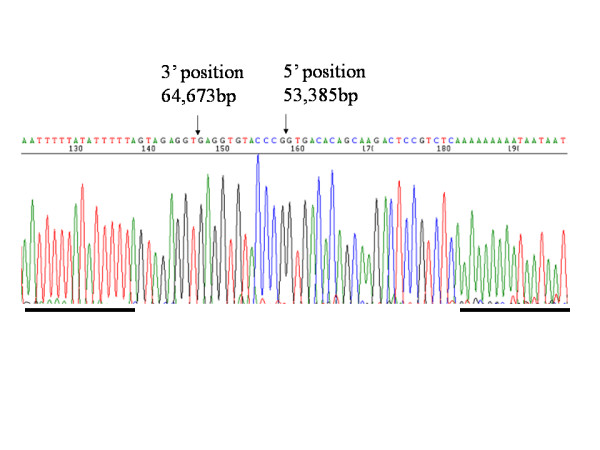
**Sequence of deletion breakpoint in the NDI family**. 5'and 3' breakpoints at positions 53,385 and 64,673 bp, respectively, relative to the published sequence (GenBank U52112.2) are indicated with arrows. The sequence is shown in antisense orientation. There is an insertion of the "GAGGTGTACCC" sequence in the break point. Bold lines indicate palindromic sequence which could cause the interstitial deletion.

Genomic DNA from patients and available family members was subjected to PCR analysis. We first used the primer pair (additional file 1) that amplifies the genomic region between exon 1 and exon 3 of *AVPR2*, and no PCR products were amplified from DNA of IV-2 and IV-4 (data not shown). Therefore, we hypothesised that a large sequence around the *AVPR2 *gene was deleted and performed PCR using the primers listed in the additional file 1. After narrowing the deleted region, we designed the primer pair AVPR2-GAP4 and obtained 386-bp PCR products for III-2, IV-2, III-3, III-4, II-4, III-10, and IV-4 (Figure 1), suggesting that these seven family members carry a large deletion that covers *AVPR2 *and part of *ARHGAP4*. The AVPR2exon1 and AVPR2exon2 primer pairs amplify the genomic region of *AVPR2 *exon 1 and exon 2, respectively. PCR was successful with DNAs from III-2, II-5, III-9, III-10, and IV-3, indicating that III-2 and III-10 are asymptomatic female carriers of the deletion. The physical map and identified deleted regions are shown in Figure 2. The deleted region includes all of the *AVPR2 *gene locus and approximately half of the *ARHGAP4 *gene. Sequencing of the 386-bp product from IV-4 revealed that the 5' and 3' break points were at positions 53,385 and 64,673 bp, respectively, relative to the published sequence (GenBank U52112.2) with an insertion of the GGGTACACCTC sequence in the break point (Figure 3).

**Table 1:** basal urine osmolality and plasma osmolality

	Basal urine osmolality	Urine osmolality response to AVP or dDAVP	Plasma osmolality
IV-1	102	102→171	NA
IV-2	30	30→26	NA
IV-4	100	100→125	308

NA; data not available

### Characteristics of immune function

We evaluated the immune function of 3 family members lacking *ARHGAP4*. None of the patients in the present study had a clinical history of recurrent or opportunistic infections. Differential leukocyte counts were within normal range (data not shown), however, a reduction of CD4+ cells was observed in III-4, and a reduction of CD8+ cells was observed in IV-4 (Table 2). Normal levels of CD3+CD4+CD45RA+ and CD3+CD4+CD45RO+ T cells were observed [18] and T cell receptor excision circles (TREC) of whole blood were within normal range in three patients tested. Serum IgG levels were relatively low in adult patients with slight reduction of CD19+ cells in III-3 and III-4. However differentiation of B cells using IgD, IgM and CD27 was not different from normal controls (data not shown).

**Table 2:** Immunological evaluation of NDI patients

	III-3 32 yr	III-4 29 yr	IV-4 5 yr	Normal range (adult)	Normal range (children)
CD3+ [%]	74.3	68.9	85.9	55–78	55–83
CD4+/CD3+ [%]	54.9	17.7	69.6	28–57	27–53
CD8+/CD3+ [%]	34.5	61.8	14	10–39	19–34
CD4/CD8	1.6	0.3	5	1.0–3.6	0.9–2.6
CD19+ [%]	5.2	5.7	6.7	6–19	10–31

IgG (mg/dl)	810.0	741.0	754.0	870–1700	600–1450
IgA (mg/dl)	147.0	72.0	105.0	110–410	39–233
IgM (mg/dl)	59.0	127.0	72.0	33–190	69–268

TREC/μgDNA	1.9 × 10^2^	2.8 × 10^2^	1.6 × 10^3^	3.4 ± 3.6 × 10^2^	3.5 ± 2.8 × 10^3^

The normal range of the lymphocyte subpopulation was lower and the upper confidence limit at a confidence level of 0.95. The normal range of IgG, IgA, and IgM are calculated as mean - 2SD (lower) and mean + 2SD (upper). The normal range of TREC is expressed as mean ± SD.

### Microarray analysis

We examined the gene expression profiles of peripheral blood mononuclear cells (PBMCs) from subjects lacking *ARHGAP4 *(III-3 and III-4) and 6 healthy male volunteers (age, 23–39 years). Transcripts satisfying all of the following criteria were identified as up- or down-regulated by microarray analysis: (1) transcripts that were expressed in at least 2 of 8 samples, (2) transcripts that showed statistically significant differences between subjects lacking *ARHGAP4 *and, controls (Welch *t*-test, q < 0.05; multiple tests were corrected by using the Benjamini and Hochberg false discovery rate [19]), and (3) transcripts that showed an average increase/decrease of more than 1.5-fold. Transcripts of *ARHGAP4 *in PBMCs in III-3 and III-4 were absent, whereas strong expression was observed in all of the controls. Up- and down-regulated genes in PBMCs lacking *ARHGAP4 *are listed in Table 3. We hypothesized that lack of *ARHGAP4 *could be compensated for by upregulation of other RhoGAP family members. Fifty-seven transcripts belonging to the RhoGAP family were included in the microarray analysis, and 25, including *ARHGAP4 *were expressed in at least 2 of 8 samples. Expression levels of the transcripts other than *ARHGAP4 *are shown in Table 4. None of the transcripts of the RhoGAP family members were statistically significantly up/down regulated in the PBMCs.

**Table 3:** Up/down regulated genes in PBMC lacking ARHGAP4

Gene Name	Description	^a^Fold change	^b ^q value	^c^Accession
Up-regulation				

PLK3	Polo-like kinase 3	4.35	0.0402	NM_004073
NHSL2	NHS-like 2	2.38	0.0453	NM_001013627
PTAFR	Platelet-activating factor receptor	2.27	0.0288	NM_000952
KLHL26	Kelch-like 26	1.99	0.0272	NM_018316
MAPKAPK2	Mitogen-activated protein kinase- activated protein kinase 2	1.96	0.0442	NM_032960
CKAP4	Cytoskeleton-associated protein 4	1.85	0.0272	NM_006825
CYB5R3	Cytochrome b5 reductase 3	1.82	0.0328	NM_007326
ZC3H12A	Zinc finger CCCH-type containing	1.81	0.0428	NM_025079
FURIN	Furin	1.79	0.0448	NM_002569
GRN	Granulin	1.76	0.0453	NM 002087
PPOX	Protoporphyrinogen oxidase	1.75	0.0307	NM_000309
NQO2	NAD(P)H dehydrogenase, quinone 2	1.74	0.0453	NM_000904
DPH3	Diphthamide biosynthesis protein 3	1.70	0.0307	NM_206831
PGD	Phosphogluconate dehydrogenase	1.68	0.00562	NM_002631
RIPK5	Receptor interacting protein kinase 5	1.66	0.0386	NM_015375
CD14	CD14 molecule	1.62	0.0272	NM_000591
PGD	Phosphogluconate dehydrogenase	1.57	0.0272	NM_002631
Down-regulation				
TUSC4	Tumor suppressor candidate 4	0.63	0.0453	NM_006545
PLEKHG4	Pleckstrin homology domain containing, family G (with RhoGef	0.62	0.0272	NM_015432
SFXN1	Sideroflexin 1	0.61	0.0272	NM_022754
GNRH1	Gonadotropin-releasing hormone 1	0.60	0.0328	NM_000825
PPP1R3E	Protein phosphatase 1, regulatory	0.60	0.0383	XM_940069
MC1R	Melanocortin 1 receptor	0.56	0.0272	NM_002386
LRCH4	Leucine-rich repeats and calponin homology (CH) domain containing 4	0.55	0.0411	NM_002319
LOC642787	Hypothetical protein LOC642787	0.44	0.0288	XM_926202
SGPP2	Sphingosine-1-phosphate phosphotase	0.09	0.0307	XM_938742
BNIPL	BCL2/adenovirus E1B 19 kD	0.06	0.0226	NM_138278
ARHGAP4	Rho GTPase activating protein 4	0.00	0.000567	NM_001666

^a^The fold change was determined by calculating the ratio of global normalized signals from the PBMCs of NDI patients to those from the PBMCs of normal subjects. ^b^P values corrected by the Benjamini and Hochberg false discovery rate, and expressed as q values ^c^GenBank accession numbers

**Table 4:** RhoGAP family gene expressions in PBMC lacking *ARHGAP4*

Gene Name	Description	^a^Fold change	^b^q value	^c^Accession
ARHGAP1	Rho GTPase activating protein 1	0.83	0.473	NM_004308
ARHGAP9	Rho GTPase activating protein 9	1.23	0.473	NM_032496
ARHGAP10	Rho GTPase activating protein10	1.01	0.914	NM_024605
ARHGAP12	Rho GTPase activating protein 12	1.03	0.655	NM_018287
ARHGAP17	Rho GTPase activating protein 17	0.67	0.473	NM_018054
ARHGAP19	Rho GTPase activating protein 19	1.16	0.473	NM_032900
ARHGAP21	Rho GTPase activating protein 21	0.98	0.655	NM_020824
ARHGAP24	Rho GTPase activating protein 24	1.33	0.891	NM_001025616
ARHGAP25	Rho GTPase activating protein 25	0.90	0.565	NM_001007231
ARHGAP25	Rho GTPase activating protein 25	0.93	0.83	NM 014882
ARHGAP27	Rho GTPase activating protein 27	0.81	0.473	NM_199282
ARHGAP30	Rho GTPase activating protein 30	1.16	0.473	NM_001025598
ARHGAP30	Rho GTPase activating protein 30	0.86	0.565	NM_181720
BM046	Rho GTPase activating protein 15	0.92	0.655	NM 018460
CDGAP	Cdc42 GTPase-activating protein	0.86	0.891	NM 020754
CENTD2	Centaurin, delta 2	1.26	0.473	NM_139181
CENTD3	Centaurin, delta 3	1.12	0.655	NM_022481
GMIP	GEM interacting protein	1.17	0.473	NM_016573
MYO9B	Myosin IXB	1.20	0.473	NM_004145
OCRL	Oculocerebrorenal syndrome of Lowe	1.00	0.655	NM_000276
PIK3R1	Phosphoinositide-3-kinase, regulatory	0.94	0.571	NM_181504
PIK3R2	Phosphoinositide-3-kinase, regulatory subunit 2 (beta)	0.86	0.655	NM_005027
RACGAP1	Rac GTPase activating protein 1	0.83	0.473	NM 013277
RALBP1	RalA binding protein 1	0.84	0.565	NM_006788

^a^Fold change was determined by calculating the ratio of global normalized signals from PBMC of NDI patients to that of normal subjects. ^b^P values corrected by the Benjamini and Hochberg false discovery rate, and expressed as q values ^c^GenBank accession number

## Discussion

In this study, we identified an extended Japanese family with X-linked NDI whose affected and carrier members have an 11.2-kb deletion and 11-bp insertion that leads to complete loss of the *AVPR2 *gene and part of the *ARHGAP4 *gene.

X-linked congenital NDI is caused by loss of or decreased function of AVPR2, and large deletions that lead to complete loss of *AVPR2 *and parts of the neighboring genes *ARHGAP4 *and L1CAM [9,11,15] have been reported.*ARHGAP4 *is expressed at high levels in hematopoietic cells. Because of the predominant expression pattern in hematopoietic cells, Schoneberg *et al *[11] performed immunologic analysis of blood from an NDI patient with complete loss of the *AVPR2 *gene and most of *ARHGAP4*. They observed a slight reduction in numbers of CD4+ cells and normal white blood cell counts, proliferation, cytokine and immunoglobulin production, respiratory burst, and phagocytosis. In addition to the patients in the present study, five NDI patients with partial deletion of *ARHGAP4 *have been reported [9,11,15], and none showed clinical signs of immunodeficiency. However, Broides *et al*. reported a pediatric NDI patient lacking *AVPR2 *and all of *ARHGAP4 *who showed immunodeficiency characterised by a marked reduction in the number of circulating CD3+ cells and an almost complete absence of CD8+ cells [17]. In this patient, the deleted region included 1.4 kb of intron 1, the first exon of *ARHGAP4 *and a 2.8 kb sequence between *ARHGAP4 *and *ARD1A*; this sequence is highly conserved across species, and Broides *et al*, speculated that this conserved region was the cause of the immunodeficiency phenotype observed in their patient. Hematologic tests of our patients showed a reduction of CD4+ cells in III-4, a reduction of CD8+ cells in IV-4, and a slight reduction of serum IgG levels in adult patients, but none of our patients showed susceptibility to infection. Taken together with the results of previous studies, our present results suggest that ARHGAP4 plays some role in lymphocyte differentiation, but does not cause the immunodeficiency phenotype. ARHGAP4 belongs to the RhoGAP family which enhances intrinsic GTPase activity. RhoGTPases are critical regulators of many cellular activities, such as motility and proliferation. We used the microarray technique to examine the possible effects on gene expression patterns in PBMCs by lacking *ARHGAP4*; several up-/down- regulated genes were identified. Our microarray study has several limitations. Firstly, it should be noted that microarray analysis is a screening method for detecting genes that may be regulated. Bosotti *et al*., reported that genes detected as differentially expressed by microarray platforms were also found to be differentially expressed by real-time quantitative PCR, although differences in the magnitudes of individual expression ratios were observed. [20] Secondly, PBMCs comprise a heterogeneous population of cells, and the possibility remains that the gene expression changes observed may merely reflect changes in the population of cells. However, because these patients lack *ARHGAP4 *gene expression in all tissues and cells, it is likely that gene expression changes may be detected even in the heterogeneous cell populations. Gene expression profiling of PBMCs lacking *ARHGAP4 *revealed that the expression of RhoGAP family genes was not influenced greatly by the lack of *ARHGAP4*, suggesting that loss of *ARHGAP4 *expression is not compensated for by other gene family members.

In the present study, one girl (IV-1) was affected by NDI. Female carriers do not usually show symptoms of NDI as observed in IV-2 and IV-4, but a small proportion of female carriers develop varying degrees of polyuria and polydipsia that can be explained by skewed inactivation of the X chromosome containing the functional *AVPR2 *allele. Although genetic testing has not been done for IV-1, it is very likely that she is a carrier of the deletion and that skewed X inactivation caused her symptoms.

A recent study showed that ARHGAP4 inhibits the migration of NIH/3T3 cells and the outgrowth of hippocampal axons and that the N-terminus and C-terminus of ARHGAP4 play different but essential roles in the potent inhibition of cell and axon motility [21]. The patients in the present study lack the C-terminus of ARHGAP4 (amino acids 515–965 according to the reference sequence NM_001666), but none of them showed clinical signs of immunodeficiencies or neurologic disorders. Further studies are needed to elucidate the role of ARHGAP4 in immune function and neuronal development.

## Conclusion

Our study indicated that loss of ARHGAP4 expression is not compensated for by other family members. ARHGAP4 may play some role in lymphocyte differentiation but partial loss of ARHGAP4 dose not result in clinical immunodeficiency.

## Methods

### Patients and mutation detection

The pedigree of the Japanese NDI family is shown in Figure 1. A full verbal and written explanation of the study was given to family members, and informed consent for the genetic study was obtained from members who participated in this study. Informed consent for the children was provided by their parents. This study was approved by the Committee of Ethics of the University of Tsukuba and was performed in accordance with the ethical standards of the 1964 Declaration of Helsinki. DNA was extracted from peripheral blood leukocytes collected in ethylenediaminetetraacetic acid (EDTA). To identify the deleted region around *AVPR2*, primers were designed according to the published sequence (GenBank accession number U52112.2) with Primer3 software [22]. The primer sequences are listed in the additional file 1. Sequencing was performed with the Big Dye Terminator kit (Applied Biosystems, Foster City, California, USA) on an ABI PRISM 3100 DNA Sequencer (Applied Biosystems).

### Immunological analysis

Peripheral blood samples from III-3, III-4 and IV-4 in Figure 1 were labeled with monoclonal antibodies according to the manufacturer's instructions and analysed with a FACSCalibur system (BD Biosciences, San Jose, California, USA). Monoclonal antibodies were purchased from BD Biosciences or Beckman-Coulter (Fullerton, California, USA). Quantification of TRECs was performed as described previously [23]. In brief, total DNA was isolated from 100 μl of peripheral blood with the use of a QIAamp DNA Micro Kit (Qiagen, Hilden, Germany) according to the manufacturer's instructions. The DNA concentration was determined with a GeneQuant pro system (GE Healthcare Bio-Sciences Corp. Piscataway, New Jersey, USA). Quantitative real-time polymerase chain reaction (PCR) for δRec-ψJα sjTRECs was performed with the following primers and probe: δRec primer, 5'-TCGTGAGAACGGTGAATGAAG-3', ψJα primer, 5'-CCATGCTGACACC'TCTGGTT-3', and δRec probe, FAM-5'-CACGGTGATGCATAGGCACCTGC-3'-TAMRA. As an internal control, *RNase P *genes were amplified in each sample tested. In each experiment, serial dilutions (10^9^, 10^8^, 10^7^, 10^6^, 10^5^, 10^3^, 10^1^, and 10^0^) of the subcloned sjTREC-plasmid (pCR4-TOPO, Invitrogen Corp., Carlsbad, California, USA) and RNase P-plasmid were used as standards for absolute quantification of TRECs and RNase P copies. Each 20-μl reaction contained 1 μl DNA, and the final concentration of each component for TREC-PCR was as follows: 2× TaqMan Universal PCR Master Mix (Applied Biosystems), 500 nM of each primer, and 250 nM TaqMan probe. The final concentration of each component for RNase P-PCR was as follows: 2× TaqMan Universal PCR Master Mix and 20× TaqMan RNase P Primer Probe (VIC dye) Mix (Applied Biosystems). PCR conditions were 50°C for 2 minutes, 95°C for 10 minutes, and 40 cycles at 95°C for 15 seconds and 60°C for 1 minute. Experiments were performed and analysed with an ABI PRISM 7300 Sequence Detection System (Applied Biosystems). Results were extrapolated to the number of TRECs per μg DNA and that of RNase P copies per μg DNA. To confirm that the DNA yields were adequately purified, TRECs were also normalised to copies of RNase P genomic DNA.

### Microarray analysis

PBMCs from III-3, III-4, and six healthy male volunteers (age, 23–39 years) were purified with a Ficoll-Paque™ gradient (GE Healthcare Bio-Sciences Corp.). RNA from PBMCs was extracted with an RNeasy Mini Kit (Qiagen) according to the manufacturer's instructions. cRNA was synthesized with an Illumina^® ^RNA Amplification Kit (Ambion, Austin, Texas, USA) according to the manufacturer's instructions. In brief, 500 ng total RNA from PBMCs was reverse transcribed to synthesise first and second strand cDNA and purified on spin columns, and in vitro transcription was performed to synthesise biotin-labeled cRNA. A total of 1500 ng biotin-labelled cRNA was hybridised to a Sentrix Human-6 Expression BeadChip version 2 (Illumina, San Diego, California, USA) at 55°C for 18 hours. The hybridised BeadChip was washed and labelled with streptavidin-Cy3 (GE Healthcare Bio-Sciences Corp.) and then scanned with the Illumina BeadStation 500 System (Illumina). The scanned image was imported into BeadStudio (Illumina) for analysis. Forty-eight thousand transcripts representing six whole-genome samples can be analysed on a single BeadChip. We included at least two technical replicates (i.e., the same cRNA samples) for each BeadChip.

Background-corrected values for each probe on the BeadChip array were extracted using BeadStudio version 1.5.1.3 (Illumina). This is based on the average of negative control genes and is called the method of background normalization by Illumina. These extracted values were exported to the software GeneSpring version 7.3.1 (Silicon Genetics, Redwood, CA), and per chip and per gene normalization were performed. The statistical significance of the microarray data was calculated using the Welch *t*-test, and multiple tests were corrected by the Benjamini and Hochberg false discovery rate [19]. Significance was defined as q < 0.05.

## Competing interests

The authors declare that they have no competing interests.

## Authors' contributions

MF, KH, RK, KK and SS carried out molecular genetic study, participated in the study design and coordination and wrote the draft of the manuscript. KI, YM and SN carried out immunologic studies. EN and TA participated in the design of the study and performed the statistical analysis. All authors read and approved the final manuscript.

## Pre-publication history

The pre-publication history for this paper can be accessed here:


